# Bidirectional brain-heart communication in coronary heart disease: from coronary plaques to central autonomic networks

**DOI:** 10.3389/fnins.2026.1942262

**Published:** 2026-09-09

**Authors:** Qi Chen, Jing Chen, Xiaoguang Chen, Gaofei Yan, Pingping Niu, Shujuan Liu, Yuanshen Zhou

**Affiliations:** 1 Department of Cardiology, Guangdong Provincial Hospital of Traditional Chinese Medicine, The Second Affiliated Hospital of Guangzhou University of Chinese Medicine, Guangzhou, China; 2 Department of Cardiovascular Disease, The First Affiliated Hospital of Guangzhou University of Chinese Medicine, Guangzhou, China; 3 Guangdong Clinical Research Academy of Chinese Medicine, Guangzhou, Guangdong, China; 4 Guangdong Provincial People’s Hospital, Guangdong Academy of Medical Sciences, Guangdong Cardiovascular Institute, Southern Medical University, Guangzhou, China

**Keywords:** brain–heart axis, central autonomic network, coronary heart disease, mental stress-induced myocardial ischemia, neuroinflammation

## Abstract

Coronary heart disease (CHD) is commonly conceptualized as a disorder of atherosclerotic plaque burden, myocardial oxygen supply-demand mismatch and thrombosis. This framework remains indispensable, yet it does not fully explain stress-induced ischemia, persistent symptoms in the absence of obstructive lesions, or neuropsychiatric and cognitive sequelae after acute coronary events. Evidence increasingly indicates that CHD is embedded in a bidirectional brain-heart axis in which central autonomic, neuroendocrine, immune, vascular, metabolic, sleep-circadian and extracellular vesicle-mediated signals interact over time. In the brain-to-heart direction, psychological stress and altered activity within central autonomic networks may promote sympathetic activation, vagal withdrawal, hypothalamic-pituitary-adrenal signaling, endothelial dysfunction, platelet activation, coronary microvascular dysregulation and inflammatory acceleration of atherosclerosis. Mental stress-induced myocardial ischemia is a clinically relevant example of this top-down pathway and has been associated with adverse outcomes in patients with established CHD. In the heart-to-brain direction, coronary ischemia and myocardial infarction may affect brain function through visceral afferent signaling, systemic and neuroinflammation, cerebral hypoperfusion, blood-brain barrier disruption and cardiac extracellular vesicles. These processes may contribute to depression, anxiety, cognitive impairment and altered interoceptive control after acute coronary events, thereby creating feedback loops that can worsen cardiovascular risk. This review critically synthesizes clinical, observational, neuroimaging, and experimental evidence concerning CHD as a bidirectional brain-heart axis disorder. We distinguish established clinical associations from emerging mechanistic pathways, highlight major methodological limitations and unresolved controversies, and discuss the current level of evidence supporting candidate biomarkers and therapeutic strategies. We argue that future CHD management should complement plaque- and ischemia-oriented strategies with assessment of autonomic function, mental health, inflammatory tone, sleep-circadian biology, cerebrovascular health and neuroimmune signaling. Such integration may help identify patients whose risk is driven not only by coronary anatomy, but also by maladaptive brain-heart communication.

## 1 Introduction

Coronary heart disease (CHD) remains one of the leading causes of morbidity and mortality worldwide. In contemporary cardiology, CHD is usually understood through coronary atherosclerosis, plaque rupture or erosion, epicardial stenosis, coronary microvascular dysfunction, thrombosis and myocardial ischemia (Byrne et al., 2023; Vrints et al., 2024). This framework has driven major advances in lipid lowering, antithrombotic therapy, revascularization, anti-inflammatory treatment and secondary prevention. Recent guidelines for chronic coronary disease and chronic coronary syndromes continue to emphasize anatomical disease, ischemic burden, risk-factor control, lifestyle intervention and long-term prevention (Virani et al., 2023). Yet many clinical observations are difficult to explain using coronary anatomy alone. Some patients develop ischemia during emotional stress without equivalent exercise-induced ischemia (Ramachandruni et al., 2006); some experience recurrent symptoms despite non-obstructive coronary arteries (Kunadian et al., 2020); some develop depression, anxiety or cognitive decline after myocardial infarction (Johansen et al., 2023; Levine et al., 2025); and some show persistent cardiovascular vulnerability even when low-density lipoprotein cholesterol, blood pressure and revascularization targets appear well-managed (Guedeney et al., 2019).

These observations point toward a broader model in which CHD is not merely a disease of the coronary artery wall or myocardium, but also a disorder of bidirectional brain–heart communication. The nervous system continuously monitors and regulates cardiovascular function through autonomic, neuroendocrine and behavioral pathways (Dampney, 2016). Conversely, the diseased heart and coronary circulation signal back to the brain through vagal and spinal afferents, inflammatory mediators, metabolic changes, vascular dysfunction and extracellular vesicles (Testai et al., 2024; Yadav et al., 2026). The result is a dynamic system rather than a one-way causal chain. The brain can shape coronary risk, ischemic susceptibility and plaque biology, while CHD can reshape neural circuits involved in emotion, interoception, cognition and autonomic control. Notably, the recently described triple-node heart-brain neuroimmune loop after myocardial infarction was demonstrated in experimental mouse models and has not yet been independently replicated or validated as a functional pathway in human CHD (Yadav et al., 2026).

The purpose of this narrative review is to critically synthesize evidence on bidirectional brain-heart communication in CHD, including atherosclerotic coronary disease, myocardial ischemia, and myocardial infarction. We focus on neural, autonomic, neuroendocrine, inflammatory, metabolic, vascular, sleep-circadian, and extracellular vesicle-mediated pathways, while distinguishing established clinical findings from observational, experimental, and hypothesis-generating evidence. Particular attention is given to conflicting results, causal limitations, and barriers to clinical translation.

## 2 Literature search and review methodology

This narrative review synthesized clinical and experimental evidence concerning bidirectional brain-heart communication in CHD. PubMed/MEDLINE, Embase, and Web of Science were searched from database inception to 30 June 2026. Reference lists of relevant reviews, guidelines, consensus statements, and key original studies were also screened to identify additional publications. Search terms combined CHD-related concepts, including “coronary heart disease,” “coronary artery disease,” “myocardial ischemia,” and “myocardial infarction,” with terms related to brain–heart communication, including “brain–heart axis,” “central autonomic network,” “autonomic dysfunction,” “mental stress-induced myocardial ischemia,” “neuroinflammation,” “cerebral perfusion,” “cognition,” “depression,” “sleep,” “extracellular vesicles,” “cardiac rehabilitation,” and “neuromodulation.” Search terms were adapted to the indexing system and syntax of each database. Studies were eligible if they examined mechanisms, clinical manifestations, biomarkers, prognostic associations, or therapeutic implications relevant to brain–heart communication in CHD or directly related experimental models. Randomized trials, observational studies, neuroimaging studies, translational investigations, animal studies, and mechanistic studies were considered. Studies without a clear relationship to CHD or brain–heart communication, duplicate publications, conference abstracts lacking sufficient data, and non-retrievable full-text articles were excluded. Only English-language publications were included. Eligibility criteria were defined according to the scope of the review before final study selection and were applied consistently during title/abstract screening and full-text assessment. The final 93 studies represented all articles that fulfilled these eligibility criteria after full-text assessment; studies were not selected on the basis of the direction, statistical significance, or perceived favorability of their findings. Accordingly, studies reporting positive, negative, null, or inconsistent associations were retained when they were otherwise relevant to the predefined review questions. This approach was intended to reduce selective inclusion of evidence supporting a particular brain–heart mechanism or clinical interpretation.

Titles and abstracts were screened, followed by full-text assessment of potentially eligible articles. During full-text assessment, exclusion was based on prespecified reasons, including lack of direct relevance to CHD or myocardial ischemia, lack of direct relevance to brain-heart communication, insufficient mechanistic or clinical information, conference-only publication with insufficient data, unavailable full text, duplicate or overlapping publication, or other clearly documented eligibility concerns. The numbers of articles excluded for each reason are reported in Supplementary Figure 1 to provide an explicit accounting of the transition from 244 full-text articles to the final 93 included studies.

Evidence was synthesized according to study design, biological pathway, population, and clinical relevance. Randomized and replicated prospective human studies were given greater weight for clinical conclusions, whereas observational and neuroimaging studies were interpreted as associative. Animal and mechanistic studies were used primarily to support biological plausibility. To minimize overrepresentation of supportive findings, synthesis was organized around predefined biological and clinical domains rather than around whether individual studies supported a proposed hypothesis. Within each domain, studies addressing comparable questions were considered together, and the consistency, direction, and strength of the evidence were evaluated in the context of study design, sample size, population characteristics, exposure or intervention definition, outcome assessment, and methodological limitations. Greater interpretative weight was assigned to randomized studies, adequately powered prospective cohorts, replicated human findings, and results supported by convergent evidence across independent populations or methodological approaches; isolated, small, retrospective, or exploratory findings were interpreted more cautiously.

Conflicting findings, methodological heterogeneity, and limitations in translating experimental evidence to human CHD were considered during synthesis. Conflicting evidence was identified when studies addressing the same or closely related biological or clinical question reported materially different directions or magnitudes of association. Such discrepancies were not resolved by preferentially selecting one result; instead, potentially explanatory differences in study design, patient characteristics, CHD phenotype or severity, sample size, timing of measurements, definitions of exposures and outcomes, analytical methods, and experimental versus clinical context were examined. Where inconsistency remained unexplained, the evidence was explicitly characterized as mixed, inconsistent, or insufficient rather than interpreted as supporting a definitive conclusion. Thus, mechanistic coherence was considered supportive but was not used to override discordant human evidence. No meta-analysis was performed. The literature identification and selection process is shown in Supplementary Figure 1.

## 3 Conceptual framework: CHD as a bidirectional brain–heart axis disorder

**FIGURE 1:**
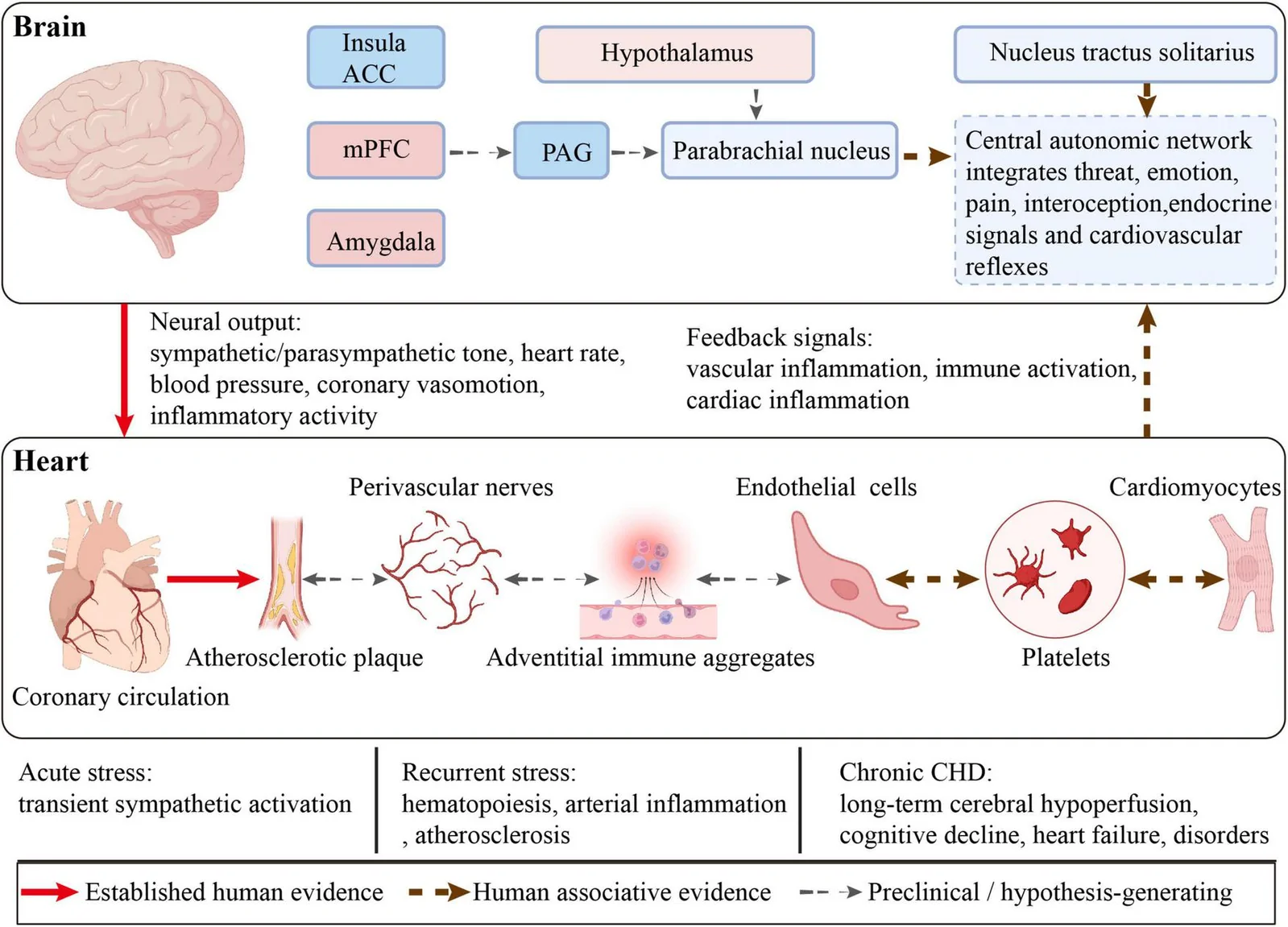
Bidirectional brain–heart axis model in coronary heart disease. This diagram places coronary heart problem (CHD) in a bidirectional brain-heart feedback system rather than as a disease of the internal heart wall. Stress-related, interoceptive, endocrine, nociceptive, cardiovascular reflex signals reach the central autonomic network, including cortical, limbic, hypothalamic and brainstem circuits, and affect coronary vulnerability through autonomic, hemodynamic, endothelial, platelet, microvascular and inflammatory processes. In the opposite direction, ischemic myocardium and diseased heart vessels send afferent neural and humoral signals to the brain via inflamed plaques, perivascular nerves, endothelium and immune cells, platelets, cardiomyocytes, and circulating mediators. The model focuses on a feedback loop between acute stress, vascular inflammation, hypoperfusion, cognitive impairments, heart failure and neuropsychiatric disorders. Solid, dashed, and dotted arrows indicate comparatively well-supported human evidence, predominantly associative human evidence, and preclinical or hypothesis-generating evidence, respectively. Arrowheads indicate the proposed direction of signal flow, and line style reflects evidence maturity rather than definitive causality.

A useful model of the CHD brain-heart axis requires three assumptions (Figure 1). First, the heart is regulated by distributed neural networks rather than by isolated autonomic reflexes. The central autonomic network includes cortical and subcortical structures such as the insula, anterior cingulate cortex, medial prefrontal cortex, amygdala, hypothalamus, periaqueductal gray, parabrachial nucleus, nucleus tractus solitarius and ventrolateral medulla. These regions integrate threat, emotion, pain, interoception, endocrine signals and cardiovascular reflexes (Beissner et al., 2013; Benarroch, 1993; Sklerov et al., 2019; Thayer and Lane, 2009). Their output influences sympathetic and parasympathetic tone, heart rate, blood pressure, vascular resistance, coronary vasomotion and inflammatory activity.

Second, the coronary circulation is not a passive target of neural output. Atherosclerotic plaques, perivascular nerves, adventitial immune aggregates, endothelial cells, platelets and cardiomyocytes produce signals that feed back to the nervous system (Mohanta et al., 2023). Neuroimmune cardiovascular interfaces in the arterial adventitia have been proposed as structural hubs in which vascular inflammation, peripheral nerves and immune cells communicate (Mohanta et al., 2022, 2023). After myocardial infarction, cardiac inflammation can also be accompanied by inflammatory activation in the brain, suggesting that acute cardiac injury can propagate beyond the heart (Bascuñana et al., 2020; Thackeray et al., 2018).

Third, brain-heart communication is shaped by time. Acute stress can trigger transient sympathetic activation, coronary vasoconstriction or mental stress-induced ischemia (Yeung et al., 1991). Recurrent stress can alter hematopoiesis, increase arterial inflammation and accelerate atherosclerosis (Heidt et al., 2014). Acute myocardial infarction can initiate short-term neuroinflammatory responses, while chronic CHD may contribute to long-term cerebral hypoperfusion, cognitive dysfunction and mood disorders (Thackeray et al., 2018). Thus, the brain-heart axis should be studied across multiple temporal scales: seconds to minutes for autonomic reflexes, hours to days for inflammatory activation, months to years for plaque progression and cognitive decline.

The purpose of this narrative review is to critically synthesize evidence on bidirectional brain-heart communication in CHD, with particular emphasis on two clinically relevant and mechanistically interconnected pathways: autonomic dysfunction and inflammation-neuroinflammation. Mental stress-induced myocardial ischemia is discussed as a major clinical model of top-down brain-heart signaling. Other pathways, including neuroendocrine, metabolic, sleep-circadian, vascular, and extracellular vesicle-mediated mechanisms, are considered primarily as modifiers, downstream consequences, or emerging translational mechanisms.

## 4 Mechanistic pathways: from established evidence to emerging frontiers

Bidirectional brain-heart communication in CHD is supported by evidence of markedly different maturity. For clinical interpretation, it is therefore more useful to organize these pathways according to the strength of available evidence rather than simply by the anatomical direction of signaling. Human physiological studies, prospective cohorts, stress-provocation studies and clinical imaging have established several clinically relevant brain-heart phenotypes, whereas other mechanisms are supported by convergent but still non-causal human data or remain predominantly experimental. This evidence-based framework helps distinguish mechanisms that can already inform clinical understanding from those that primarily define priorities for translational research.

### 4.1 Clinically established mechanisms and phenotypes

Autonomic regulation provides the most direct and clinically established link between the brain and coronary circulation (Benarroch, 1993; Ferraro et al., 2022; Sklerov et al., 2019). The central autonomic network integrates emotional, cognitive, interoceptive and homeostatic information through distributed cortical, limbic, hypothalamic and brainstem regions, with the insular and midcingulate cortices emerging as relatively consistent components in human neuroimaging studies (Ferraro et al., 2022; Kleckner et al., 2017; Strigo and Craig, 2016; Taylor et al., 2016; Valenza et al., 2019). Its cardiovascular output is expressed through sympathetic and parasympathetic pathways. Increased sympathetic activity raises heart rate, myocardial contractility and blood pressure, thereby increasing myocardial oxygen demand, while also promoting coronary vasoconstriction and reducing the capacity for adaptive vascular responses (Goodwill et al., 2017; Padro et al., 2020). Vagal withdrawal further reduces cardiovascular flexibility. In patients with CHD, particularly those with impaired coronary flow reserve, this combination can lower the threshold for myocardial ischemia.

The clinical importance of autonomic dysfunction is most clearly demonstrated after myocardial infarction. Heart rate variability (HRV) and baroreflex sensitivity provide complementary measures of cardiac autonomic regulation and have long been associated with prognosis (Jarczok et al., 2022; Kaufmann et al., 2021). In the multicenter ATRAMI study, an SDNN below 70 ms and a baroreflex sensitivity below 3.0 ms/mmHg were independently associated with approximately 3.2-fold and 2.8-fold increases in cardiac mortality, respectively; when both indices were markedly depressed, 2-year cardiac mortality reached 17%, compared with 2% in patients with preserved values (La Rovere et al., 1998; Takase et al., 2024). These measurements are influenced by age, medication, respiration, rhythm and recording methodology, and much of the landmark evidence predates contemporary reperfusion and secondary prevention. They should therefore be interpreted as physiological and prognostic markers rather than stand-alone therapeutic targets. Nevertheless, the association between impaired autonomic regulation and adverse cardiovascular outcomes after myocardial infarction is well-supported.

Mental stress-induced myocardial ischemia (MSIMI) provides an especially informative clinical model of top-down brain-heart signaling. Unlike conventional exercise-induced ischemia, MSIMI frequently develops at a relatively low hemodynamic workload and reflects the convergence of autonomic activation with endothelial dysfunction, abnormal coronary vasomotion and coronary microvascular dysregulation (Mehta et al., 2022; Sullivan et al., 2018). Standardized mental stressors such as public speaking, anger recall or arithmetic tasks can induce transient myocardial perfusion defects or left ventricular dysfunction in susceptible patients. Importantly, MSIMI is associated with subsequent cardiovascular events in patients with established CHD, demonstrating that this phenotype has prognostic relevance beyond the laboratory setting (Vaccarino et al., 2021). Mental stress-related endothelial dysfunction has also been associated with adverse cardiovascular outcomes, supporting vascular reactivity as an important downstream component of the stress response (Lima et al., 2019).

Sex differences further illustrate the clinical relevance of this pathway. Younger women with recent myocardial infarction show greater susceptibility to MSIMI than age-matched men, potentially reflecting a combination of psychosocial stress burden, autonomic responses, endothelial dysfunction and coronary microvascular vulnerability (Mehta et al., 2018; Osei et al., 2024; Vaccarino et al., 2018). This observation is particularly relevant to patients with angina or ischemia and non-obstructive coronary arteries, in whom impaired coronary flow reserve, endothelial dysfunction and microvascular spasm may coexist (Del Buono et al., 2021). These findings reinforce the concept that ischemic susceptibility cannot always be inferred from epicardial stenosis alone.

Communication in the opposite direction is equally fundamental to the clinical expression of CHD. Myocardial ischemia generates visceral afferent signals that ascend through spinal and brainstem pathways to thalamic, insular and cingulate regions involved in pain, salience and autonomic regulation (Foreman et al., 2015; Gallone et al., 2020). Human imaging demonstrates that painful and silent myocardial ischemia do not produce identical cerebral responses: both can activate the thalamus, whereas painful ischemia recruits broader cortical regions involved in conscious perception and appraisal (Rosen et al., 1996). Direct stimulation studies also demonstrate that the human insula can modify cardiac autonomic responses (Chouchou et al., 2019). Thus, angina is not simply a direct readout of myocardial ischemic burden; conscious symptom perception depends on interactions between peripheral cardiac signaling and central processing.

This mechanism also helps explain why symptom experience varies substantially between patients. After an acute coronary event, heightened attention to heart rate, dyspnea or chest sensations may amplify perceived bodily threat and promote fear of exertion or avoidance (Alcántara et al., 2020; Leissner et al., 2022). Psychological distress after myocardial infarction is clinically important because depression, anxiety and trauma-related symptoms can impair medication adherence, physical activity, sleep and participation in cardiac rehabilitation (Levine et al., 2025). Recent ambulatory data further link greater cardiac event-induced post-traumatic stress symptoms with higher continuous heart rate, although the causal direction remains uncertain (Ten Brink et al., 2026). The interaction between visceral signaling, autonomic arousal and behavioral responses therefore provide a clinically relevant feedback pathway through which a cardiac event can continue to influence recovery after the acute lesion has stabilized.

Systemic inflammation after myocardial infarction represents another well-documented component of heart-brain communication. Acute myocardial injury is followed by rapid recruitment of neutrophils and monocytes and changes in circulating inflammatory mediators (Oprescu et al., 2021). This response is required for clearance of necrotic tissue and initiation of repair, whereas excessive or poorly resolved inflammation can contribute to adverse ventricular remodeling (Frangogiannis, 2014). At the same time, inflammatory mediators, altered endothelial function and changes in cerebral perfusion provide plausible routes through which acute cardiac injury can influence the brain.

The clinical neurological and psychological consequences after coronary events are also increasingly recognized. Depression and other forms of psychological distress are common after myocardial infarction and warrant active cardiovascular follow-up (Levine et al., 2025). Cognitive vulnerability is likewise clinically relevant. Longitudinal studies have associated myocardial infarction with subsequent decline in global cognition, although the magnitude and trajectory vary considerably among patients (Johansen et al., 2023; Ridha et al., 2026). These outcomes are unlikely to result from a single mechanism. Shared vascular risk factors, cerebral small-vessel disease, impaired perfusion, inflammation, sleep disturbance, depression, medication exposure and reduced physical activity may all contribute. For clinical practice, the important point is that cardiac injury can have persistent effects on mood, cognition and behavior, even though the relative contribution of individual biological pathways differs between patients.

### 4.2 Mechanisms supported by substantial but not definitive evidence

Human neuroimaging has substantially advanced understanding of the central mechanisms underlying autonomic and stress-related cardiovascular responses. Meta-analytic data consistently implicate the insular and midcingulate cortices, while the amygdala, prefrontal cortex, hypothalamus and brainstem are recruited according to the specific stressor and physiological response (Ferraro et al., 2022; Gianaros et al., 2017). Higher stress-related amygdalar activity has been associated with arterial inflammation, coronary plaque vulnerability and subsequent cardiovascular events (Dai et al., 2023; Tawakol et al., 2017). These observations support a distributed neural network through which emotional and cognitive stress may influence cardiovascular physiology. However, most imaging studies remain observational, and regional brain activity cannot yet be interpreted as evidence that dysfunction of an individual neural node directly causes coronary events.

Neuroendocrine signaling provides an additional bridge between psychological stress and cardiovascular biology. Activation of the hypothalamic-pituitary-adrenal axis and sympathetic-adrenal system alters cortisol and catecholamine signaling, with downstream effects on blood pressure, glucose metabolism, endothelial function and inflammatory regulation (Dar et al., 2019; Iob and Steptoe, 2019; Vaccarino and Bremner, 2024). Chronic stress may therefore promote a metabolic and vascular environment favorable to CHD progression. However, cortisol responses vary with circadian timing, sleep, metabolic state, medication use and individual stress reactivity (Sher et al., 2020). A single cortisol measurement cannot identify a specific brain-heart phenotype, and the independent contribution of hypothalamic-pituitary-adrenal dysregulation to CHD progression remains difficult to separate from concurrent autonomic, metabolic and inflammatory pathways.

A related mechanism links psychological stress to hematopoiesis and vascular inflammation. Experimental studies show that chronic stress can activate hematopoietic stem and progenitor cells, increase inflammatory leukocyte production and accelerate vascular inflammation (Heidt et al., 2014). Human PET studies provide convergent evidence by linking increased amygdalar activity with bone-marrow activity, arterial inflammation, coronary plaque vulnerability and cardiovascular events (Dai et al., 2023; Tawakol et al., 2017). Together, these findings support a brain-bone marrow-arterial pathway in which central stress processing may influence plaque biology through sympathetic and immune mechanisms. Nevertheless, human imaging signals remain indirect, and the complete causal sequence from altered neural activity to hematopoietic activation, plaque destabilization and clinical coronary events has not been demonstrated prospectively (Dai et al., 2023; Tawakol et al., 2017).

Stress-related endothelial and coronary microvascular responses provide another plausible intermediate mechanism. Mental stress can transiently impair endothelial function, and this response has been associated with subsequent cardiovascular events (Lima et al., 2019). In susceptible patients, sympathetic vasoconstriction may coincide with reduced nitric oxide-mediated vasodilation, abnormal microvascular reactivity and increased inflammatory signaling (Hammadah et al., 2017a; Khan et al., 2017). These effects are particularly relevant in ANOCA/INOCA phenotypes, in which symptoms and ischemia may occur despite the absence of severe epicardial stenosis (Del Buono et al., 2021). Recent data also suggest that higher resting IL-6 concentrations are associated with more severe mental stress-induced myocardial perfusion defects in women with ANOCA (Yin et al., 2026). The observation is mechanistically intriguing but requires replication before IL-6 can be considered a mediator or clinically useful biomarker of stress-induced ischemia.

The biological response of the brain after myocardial infarction is supported by a growing body of imaging and translational evidence. PET studies have demonstrated parallel inflammatory signals in the myocardium and brain after infarction, and greater myocardial inflammatory activity has been associated with adverse ventricular remodeling and increased cerebral inflammatory signals (Thackeray et al., 2018). These findings provide a mechanistic basis for systemic immune-to-brain signaling after acute cardiac injury. However, neuroinflammation imaging remains difficult to interpret because commonly used translocator protein PET signals are not specific to microglia and may also reflect astrocytes, endothelial cells or infiltrating macrophages (Guilarte et al., 2022). A direct causal pathway from post-infarction neuroinflammation to depression, cognitive decline or recurrent cardiovascular events therefore remains unproven in humans.

Cerebrovascular dysfunction may provide an additional heart-to-brain pathway. Reduced cardiac output, arterial stiffness, endothelial dysfunction, impaired cerebral autoregulation and shared vascular risk factors can all compromise cerebral perfusion (Jefferson et al., 2017; Liu et al., 2021). Impaired cerebrovascular function has been demonstrated in patients with coronary artery disease (Anazodo et al., 2015), while longitudinal cohorts have associated myocardial infarction with subsequent cognitive decline (Johansen et al., 2023; Ridha et al., 2026). The available data do not establish cerebral hypoperfusion as the sole or dominant mechanism. Instead, cognitive outcomes probably reflect interactions among vascular injury, systemic inflammation, depression, sleep disturbance and pre-existing cerebral reserve.

Sleep and circadian biology further modify these pathways. Sleep disorders can increase sympathetic activity, blood pressure and cardiometabolic burden (Tasali et al., 2025), while endogenous circadian rhythms influence platelet activation, with greater platelet responsiveness during the biological morning (Scheer et al., 2011). These processes may help explain temporal variation in coronary risk and may interact with stress, autonomic dysfunction and metabolic disease. Their importance for cardiovascular health is well-supported, but the extent to which they operate through a specific brain-heart mechanism in individual patients with CHD remains incompletely defined.

### 4.3 Emerging mechanisms and research frontiers

Neuroimmune cardiovascular interfaces represent one of the most promising mechanistic frontiers. Experimental atherosclerosis studies have identified anatomical and functional interactions among adventitial immune aggregates, peripheral nerves and the arterial wall, with disruption of components of these circuits modifying atherosclerosis progression (Mohanta et al., 2022). More recently, a triple-node heart-brain neuroimmune loop after myocardial infarction has been demonstrated experimentally, suggesting that cardiac injury may engage organized neural and immune circuits extending beyond conventional circulating inflammatory pathways (Yadav et al., 2026). These findings substantially expand the conceptual framework of brain-heart communication, but equivalent functional circuits have not yet been established in human CHD. Direct human anatomical validation, longitudinal imaging and intervention studies will be required to determine whether these pathways influence plaque progression, recurrent ischemia or post-infarction recovery.

Extracellular vesicles offer a second emerging route of molecular heart-brain communication. Cardiomyocyte-derived extracellular vesicles can carry microRNAs, proteins, lipids and other biologically active cargo. Experimental studies indicate that vesicles released after myocardial injury can reach the brain and modify neuroinflammatory or cognitive responses; specific vesicle-associated microRNAs have been implicated in these effects (Chen et al., 2026; Li et al., 2025). Human studies currently show that circulating extracellular vesicle profiles change after myocardial infarction and may reflect the severity of myocardial injury (Zarà et al., 2023). Whether cardiac extracellular vesicles cross the human blood-brain barrier in biologically meaningful quantities, target specific brain regions, or contribute directly to depression, cognitive impairment or autonomic dysfunction remains unknown.

A further priority is to move from isolated biomarkers toward integrated mechanistic phenotyping. Measures such as HRV, inflammatory markers, stress-provocation testing, coronary microvascular assessment and brain imaging each capture only one component of the brain-heart system. Future prospective studies should determine whether combinations of these measures identify reproducible patient phenotypes and, more importantly, whether such phenotypes predict differential responses to treatment. Longitudinal studies with repeated measurements before and after coronary events will be particularly important for establishing temporal directionality. The key translational question is therefore not simply whether the brain and heart communicate in CHD, but which pathways are causal, in which patients they dominate, and whether interrupting them improves clinically meaningful cardiovascular and neuropsychological outcomes.

## 5 An integrated vicious-cycle model

**FIGURE 2:**
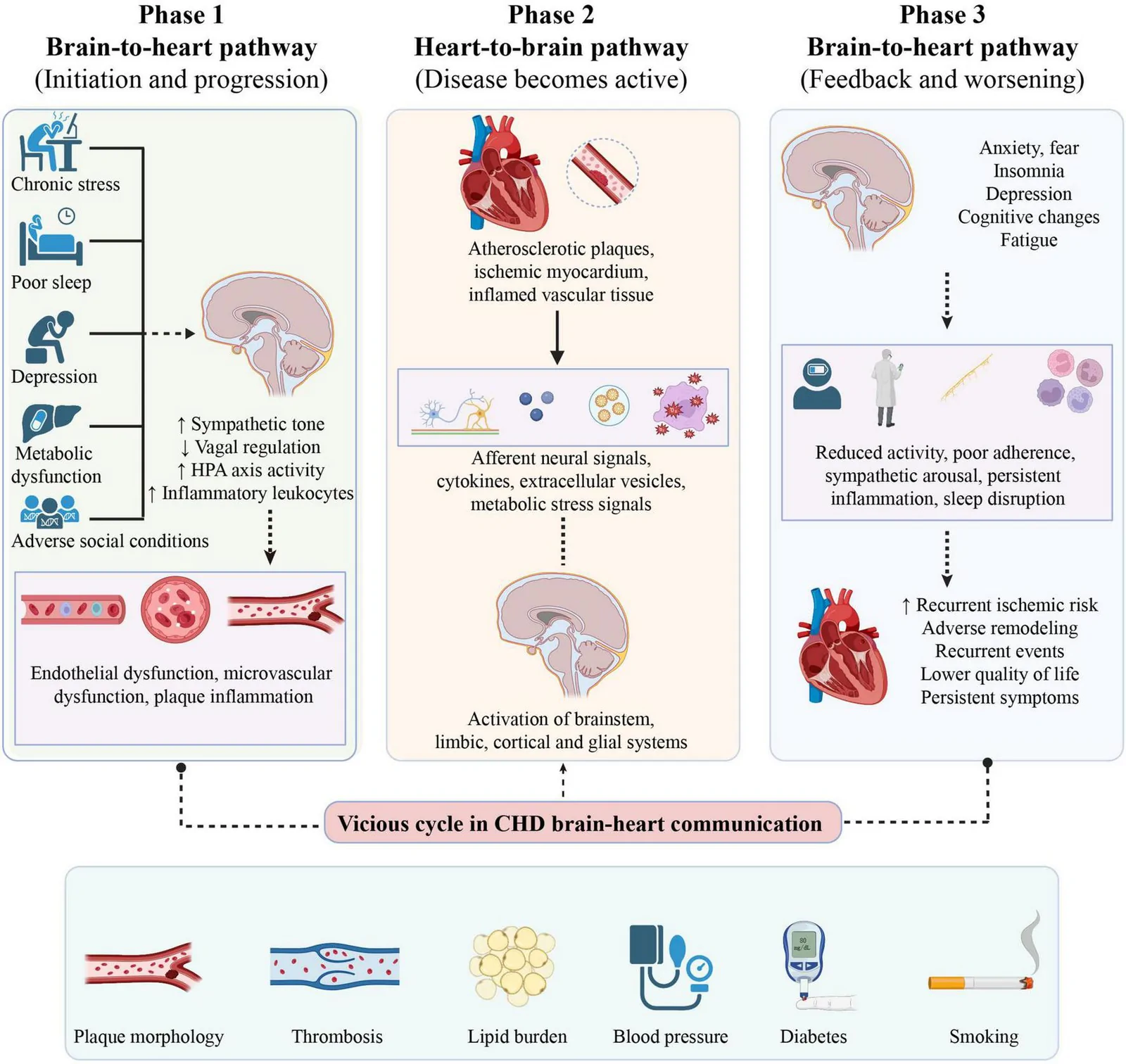
A vicious-cycle model of coronary heart disease brain-heart communication. This model illustrates how chronic stress, poor sleep, depression, metabolic dysfunction, and adverse social conditions may disrupt autonomic, endocrine, and inflammatory regulation, thereby promoting endothelial dysfunction, coronary microvascular dysfunction, and plaque inflammation. In turn, atherosclerotic plaques, ischemic myocardium, and inflamed vessels may send neural, immune, and metabolic signals to brainstem, limbic, cortical, and glial systems. Resulting anxiety, sympathetic activation, inflammation, sleep disturbance, inactivity, and poor adherence may further increase recurrent ischemia, adverse remodeling, persistent symptoms, and impaired quality of life. Conventional risk factors, including thrombosis, lipid burden, hypertension, diabetes, and smoking, remain central to this process. Solid, dashed, and dotted arrows indicate comparatively well-supported human evidence, predominantly associative human evidence, and preclinical or hypothesis-generating evidence, respectively. Arrowheads indicate the proposed direction of signal flow, and line style reflects evidence maturity rather than definitive causality. Solid arrows, well-supported human physiological/clinical evidence; Dashed arrows, human observational, associative or neuroimaging evidence; Dotted arrows, preclinical, early-translational or hypothetical pathways.

What the evidence reviewed above ultimately describes is something disquieting: a self-reinforcing loop of CHD brain-heart communication (Figure 2).

The first phase is, in many respects, where the trouble quietly begins. Chronic stress, poor sleep, depression, metabolic dysfunction, and adverse social conditions do not merely accompany one another–they begin to recast central autonomic and limbic networks in ways that compound over time. Sympathetic tone climbs. Vagal regulation retreats. The hypothalamic-pituitary-adrenal axis stirs into sustained activity, and inflammatory leukocyte production gains momentum. Slowly, this altered internal landscape accelerates endothelial dysfunction, coronary microvascular dysfunction, and atherosclerotic plaque inflammation.

In the second phase, the consequences turn visible. Atherosclerotic plaques, ischemic myocardium, and inflamed vascular tissue begin to broadcast afferent neural signals, cytokines, extracellular vesicles, and metabolic stress signals, all converging on brainstem, limbic, cortical, and glial systems. The patient now feels it: angina, dyspnea, fear, fatigue, insomnia, depression, or a creeping cognitive fog.

The third phase is where the loop closes, and it does so with troubling efficiency. Anxiety about symptoms, reduced activity, poor adherence, sympathetic arousal, persistent inflammation, and disrupted sleep collectively push recurrent ischemic risk upward. After myocardial infarction, this feedback may drive adverse remodeling, fuel recurrent events, and steadily hollow out quality of life. In chronic coronary disease, the cycle can maintain its grip on symptoms long after epicardial stenosis has been addressed.

None of this displaces the well-established mechanisms of CHD. Plaque morphology, thrombosis, lipid burden, blood pressure, diabetes, and smoking retain their central importance. What the brain-heart axis brings to the picture is a systems-level explanation for something clinicians have long found puzzling: why patients with near-identical coronary anatomy can diverge so sharply in symptoms, stress responses, inflammatory profiles, recovery trajectories, and long-term outcomes.

**FIGURE 3:**
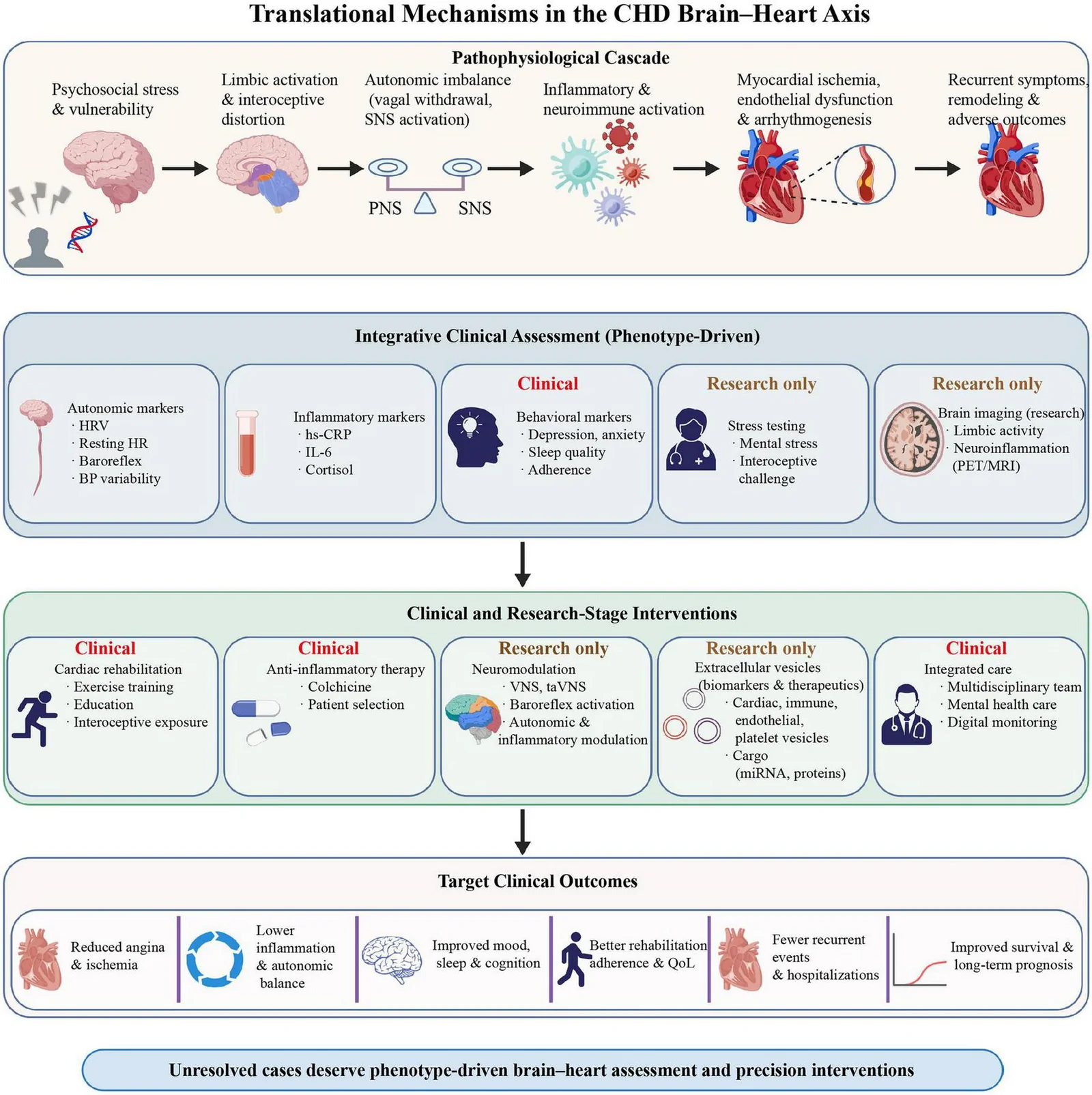
Translational mechanisms in the coronary heart disease brain-heart axis. Psychosocial stress may promote limbic, autonomic, inflammatory, and neuroimmune dysregulation, contributing to myocardial ischemia, endothelial dysfunction, arrhythmogenesis, and recurrent adverse outcomes. A phenotype-driven assessment combining autonomic, inflammatory, behavioral, stress-testing, and research imaging markers may guide targeted interventions, including cardiac rehabilitation, anti-inflammatory therapy, neuromodulation, extracellular vesicle-based strategies, and integrated multidisciplinary care, with the aim of improving symptoms, recovery, and long-term prognosis.

Taken together, these considerations support a phenotype-driven framework that links brain-heart pathophysiology with integrated clinical assessment, targeted translational interventions, and clinically meaningful outcomes (Figure 3).

## 6 Clinical and translational implications

Contemporary management of CHD appropriately prioritizes coronary anatomy, ischemic burden, lipid lowering, antithrombotic therapy, risk-factor control and, when indicated, revascularization (Virani et al., 2023). These strategies remain the foundation of care. However, they do not fully explain why some patients continue to experience angina, impaired recovery or recurrent cardiovascular vulnerability despite apparently adequate treatment of conventional coronary risk. Brain-heart mechanisms may be particularly relevant to this residual burden when symptoms are linked to emotional stress, coronary anatomy does not adequately explain ischemic symptoms, or recovery after myocardial infarction is complicated by autonomic, psychological or behavioral disturbance. The clinical value of the brain-heart framework therefore lies not in creating a new universal diagnostic pathway, but in identifying selected patients in whom established or emerging brain-heart processes may complement conventional cardiovascular assessment. A practical phenotype-to-action framework for this selective approach is summarized in Table 1.

**TABLE 1:** A practical phenotype-to-action framework for brain–heart assessment in Coronary heart disease (CHD).

Clinical scenario	What to consider	What to do now	What not to do routinely
Persistent angina unexplained by coronary anatomy	CMD, vasospasm; stress-linked symptoms	Standard ischemia/rhythm evaluation; assess CMD/vasospasm when indicated	Do not attribute symptoms to psychological factors by exclusion
Reproducible stress-linked symptoms or ischemia	MSIMI, particularly after MI	Optimize CHD care; recognize stress-related phenotype	Routine mental-stress testing or brain imaging
Post-MI impaired recovery	Depression, anxiety/PTSD, sleep disturbance, fear of exertion	Validated screening; cardiac rehabilitation; referral when indicated	Dedicated brain-heart biomarker panels
Post-MI autonomic or residual inflammatory risk	HRV/BRS; residual inflammation	Standard secondary prevention; selective HRV or hs-CRP assessment	Biomarker-guided therapy based on HRV, cytokines, or imaging alone
Experimental brain-heart phenotypes	N euroinflammation, neuroimmune signaling, EVs, neuromodulation	Research protocols	Routine clinical testing or treatment

BRS, baroreflex sensitivity; CHD, coronary heart disease; CMD, coronary microvascular dysfunction; EVs, extracellular vesicles; HRV, heart rate variability; MI, myocardial infarction; MSIMI, mental stress-induced myocardial ischemia; PTSD, post-traumatic stress disorder.

### 6.1 Established clinical actions: identifying and managing residual brain-heart risk

Brain-heart assessment should begin only after standard cardiovascular evaluation has been appropriately addressed. Coronary anatomy, conventional ischemia, arrhythmia, medication adherence, major cardiovascular risk factors and, when clinically indicated, coronary microvascular dysfunction or vasospastic disease should remain the first considerations (Del Buono et al., 2021; Kunadian et al., 2020; Virani et al., 2023). Additional brain-heart-oriented assessment is most relevant when there is a mismatch between conventional findings and the clinical phenotype.

One such phenotype is persistent or recurrent angina despite non-obstructive coronary arteries or apparently adequate revascularization. In these patients, coronary microvascular dysfunction and vasospastic disease should be considered according to established diagnostic pathways rather than attributing symptoms directly to psychological stress (Del Buono et al., 2021; Kunadian et al., 2020). Emotional stress may nevertheless provide an important clue when symptoms are reproducibly stress-related or discordant with conventional exercise testing. Mental stress-induced myocardial ischemia (MSIMI) demonstrates that emotional stress can provoke objective myocardial ischemia in susceptible patients and is associated with subsequent cardiovascular events (Vaccarino et al., 2021). This phenotype appears particularly relevant in younger women after myocardial infarction, although these findings should not be generalized to all women with CHD (Vaccarino et al., 2018). Thus, stress-linked symptoms should prompt careful cardiovascular phenotyping rather than dismissal as non-cardiac complaints.

A second clinically important setting is recovery after myocardial infarction. Depression, anxiety, trauma-related symptoms and sleep disturbance can affect medication adherence, physical activity, symptom perception, and participation in cardiac rehabilitation (Levine et al., 2021, 2025). Their recognition should therefore form part of cardiovascular follow-up rather than being considered separate from cardiac care. Brief validated depression and anxiety screening can be incorporated into early post-infarction follow-up or cardiac rehabilitation intake, with targeted assessment of post-traumatic stress and sleep disturbance when clinically indicated. Positive screening results should lead to clinical confirmation and a defined management or referral pathway (Levine et al., 2025). The objective is not to redefine CHD as a psychological disorder, but to identify modifiable factors that may interfere with cardiovascular recovery.

Cardiac rehabilitation is particularly relevant within this framework because it addresses several of these domains simultaneously. Exercise-based rehabilitation improves functional capacity and can favorably influence endothelial function and cardiovascular risk (Hambrecht et al., 2000). In patients who develop fear of exertion after an acute coronary event, supervised exercise may also restore confidence in physiological sensations such as increased heart rate and breathlessness and reduce progressive activity avoidance (Baykal Şahin et al., 2021; Johnsson et al., 2025). Psychological support, sleep assessment and individualized reintroduction to physical activity can therefore be incorporated into comprehensive rehabilitation when required, without creating a separate “brain–heart” treatment program.

Autonomic measures can provide additional physiological context, but they should not displace these established clinical actions. Reduced heart rate variability (HRV) and impaired baroreflex sensitivity (BRS) have established prognostic associations after myocardial infarction (La Rovere et al., 1998). However, HRV is affected by age, rhythm, respiration, medication exposure and recording methodology, and its predictive value for individual patients is limited (Sassi et al., 2015). Accordingly, HRV may provide supportive information in selected post-infarction or arrhythmia-prone patients, whereas BRS is more appropriately confined to specialist or research settings. Neither measure currently provides a stand-alone indication for treatment.

The practical implication is therefore relatively simple: clinicians should first optimize guideline-directed CHD care and then identify potentially modifiable contributors to residual symptoms or impaired recovery. In appropriate patients, this means evaluating coronary microvascular or vasospastic disease, recognizing reproducible stress-linked symptoms, screening for clinically important psychological or sleep disturbance, and facilitating comprehensive cardiac rehabilitation. These measures are already clinically actionable and do not require a dedicated brain-imaging or biomarker panel.

### 6.2 Selective assessments and therapies with supportive but incomplete evidence

Several additional approaches may refine brain-heart phenotyping but are not sufficiently standardized for routine use in unselected patients with CHD. Their role should therefore be determined by a specific clinical or research question rather than by broad screening.

Heart rate variability and BRS illustrate this distinction. Both provide physiologically meaningful information on autonomic regulation, and markedly impaired values after myocardial infarction have been associated with adverse outcomes (La Rovere et al., 1998). Nevertheless, methodological heterogeneity and the absence of validated treatment thresholds limit their role as independent clinical decision tools (Sassi et al., 2015). Similarly, laboratory mental-stress testing can identify MSIMI using myocardial perfusion imaging or inducible ventricular dysfunction and has provided important prognostic and mechanistic information (Vaccarino et al., 2018, 2021). At present, however, mental-stress testing should not replace conventional ischemia testing. Its most appropriate role remains in specialist centers or research protocols, particularly for patients with reproducibly stress-linked symptoms or suspected stress-sensitive ischemic phenotypes.

Inflammatory assessment occupies a similar intermediate position. Residual inflammatory risk can persist despite intensive lipid lowering and contemporary secondary prevention (Guedeney et al., 2019). Among available markers, high-sensitivity C-reactive protein (hs-CRP) is the most clinically accessible, although interpretation requires a clinically stable patient and exclusion of acute infection or other inflammatory conditions. More experimental measures, including IL-6 profiling, cortisol responses, multimarker panels and neuroinflammatory imaging, are not sufficiently standardized for routine CHD risk stratification.

Randomized trials provide evidence that inflammation itself is therapeutically modifiable in selected patients with atherosclerotic cardiovascular disease. Canakinumab reduced recurrent cardiovascular events in patients with previous myocardial infarction and elevated inflammatory risk, while low-dose colchicine reduced cardiovascular events in chronic coronary disease (Nidorf et al., 2020; Ridker et al., 2017). Conversely, more recent data in acute myocardial infarction have shown neutral results for colchicine in that setting, emphasizing the importance of patient selection, timing and background therapy (Jolly et al., 2025).

These findings should not, however, be interpreted as evidence that anti-inflammatory therapy specifically corrects brain-heart dysfunction. Current evidence does not support using HRV, BRS, a single cytokine concentration, brain imaging or another putative brain-heart biomarker alone to select anti-inflammatory or other cardiovascular therapy. Anti-inflammatory treatment should therefore follow established cardiovascular evidence and relevant guideline recommendations rather than the presence of an assumed brain-heart phenotype. Whether patients characterized by autonomic dysfunction, MSIMI or persistent inflammatory activation derive differential benefit from specific interventions remains an important question for prospective studies.

### 6.3 Translational frontiers and research priorities

Several approaches discussed within the brain-heart field remain predominantly translational and should be clearly separated from currently actionable clinical care. Among the most important are post-infarction neuroinflammation imaging, neuroimmune cardiovascular interfaces, neuromodulation and extracellular vesicle-mediated signaling.

Imaging studies provide evidence that myocardial infarction can be accompanied by inflammatory signals in the brain, raising the possibility that cardiac injury triggers coordinated peripheral and central immune responses (Thackeray et al., 2018). However, commonly used neuroinflammation imaging markers are not cell-specific, and available human studies remain small. Neuroimaging therefore cannot currently determine whether post-infarction neuroinflammation directly causes depression, cognitive decline or recurrent cardiovascular events (Guilarte et al., 2022). Similarly, experimental studies have identified neuroimmune interfaces linking adventitial immune structures, peripheral nerves and the arterial wall, but functional equivalents of these circuits have not yet been established in human CHD (Mohanta et al., 2022). These pathways are mechanistically important but currently provide hypotheses for human investigation rather than clinical targets.

Neuromodulation represents another attractive but unproven strategy. Vagus nerve stimulation and baroreflex activation can theoretically modify autonomic signaling and inflammatory reflex pathways, but most human cardiovascular experience has been obtained in heart failure rather than CHD. Randomized studies of vagus nerve stimulation in heart failure have produced heterogeneous results, while baroreflex activation has shown improvements in selected functional and biomarker outcomes without establishing efficacy against coronary ischemia or recurrent coronary events (Gold et al., 2016; Zannad et al., 2015; Zile et al., 2020). Experimental ischemia-reperfusion studies provide proof-of-concept that vagal stimulation may reduce inflammatory injury, infarct size or arrhythmic risk, but these findings have not been validated as a treatment strategy in patients with CHD (Hu et al., 2026; Zhang et al., 2026). Neuromodulation should therefore remain within clinical trials. Future CHD studies should enrich for patients with measurable target phenotypes, incorporate sham controls and demonstrate target engagement before assessing clinical outcomes.

Extracellular vesicles (EVs) are similarly promising but remain far from routine clinical application. Experimental studies suggest that vesicles released after cardiac injury can participate in heart-to-brain signaling and influence neuroinflammatory or cognitive responses (Chen et al., 2026; Li et al., 2025). In humans, however, current evidence primarily supports circulating EVs as potential markers of myocardial injury rather than as proven mediators of brain-heart communication (Zarà et al., 2023). It remains uncertain whether cardiomyocyte-derived EVs cross the human blood-brain barrier in biologically relevant quantities, show reproducible neural targeting or predict neuropsychological outcomes after myocardial infarction. EV profiling should therefore be regarded as a research tool rather than a clinical biomarker panel.

Across these emerging fields, the priority is no longer simply to demonstrate that neural, immune and cardiovascular signals are associated. Future studies need to establish temporal directionality, reproducible target engagement and incremental clinical value. Prospective studies should determine whether specific phenotypes, such as autonomic dysfunction, reproducible MSIMI or persistent inflammatory activation, identify patients who respond differently to targeted interventions. For young cardiologists, the key distinction is therefore between mechanisms that already change clinical management and mechanisms that currently define research priorities. Brain-heart assessment is most useful when it complements established CHD care and helps explain residual symptoms or risk; it should not become an additional layer of testing without an actionable clinical consequence.

### 6.4 Registered clinical studies and near-term translational priorities

Representative registered studies illustrate ongoing efforts to translate selected brain–heart-related phenotypes into prospective clinical research, although registry status and study completion should not be interpreted as evidence of clinical efficacy. At the time of the latest registry verification, PACE SETTER (NCT05925634) was evaluating individualized exercise-prescription strategies and psychological feedback during cardiac rehabilitation; Mental Stress and Myocardial Ischemia After MI (NCT04123197) had completed enrollment and follow-up of a younger post-myocardial-infarction cohort designed to examine sex differences, mechanisms, and prognosis related to mental stress-induced myocardial ischemia; GENAMI PREVENTION (NCT05619601) was listed as completed and had evaluated sex differences in secondary-prevention adherence after type 1 acute myocardial infarction; and CorMicA-PCI (NCT06854302) remained an ongoing prospective cohort study investigating mechanisms of persistent or recurrent angina after PCI using coronary physiological assessment and cardiovascular magnetic resonance imaging (Berry, 2024; Pack, 2023; Spanish Society of Cardiology, 2022; Vaccarino, 2019). These registry entries describe study objectives and status rather than validated clinical findings; therefore, they are cited as indicators of current research direction and should not be interpreted as evidence supporting routine implementation.

## 7 Conclusion and future directions

Several limitations and knowledge gaps remain. Much of the current human evidence linking the brain-heart axis to CHD is associative, and causal relationships are difficult to establish from neuroimaging, inflammatory profiling or clinical outcome studies alone (Khalil et al., 2024; Thackeray et al., 2018). Although experimental models provide mechanistic insights, their relevance to human CHD requires further validation. Another major challenge is disease heterogeneity. Stable obstructive CHD, acute coronary syndromes, myocardial infarction with non-obstructive coronary arteries, coronary microvascular dysfunction and vasospastic angina may involve distinct patterns of autonomic, neuroimmune, vascular and psychosocial regulation (Tamis-Holland et al., 2019; Vrints et al., 2024). Therefore, CHD should not be treated as a single biological entity in future brain-heart studies. Sex and gender differences also remain insufficiently characterized. The greater vulnerability of young women to mental stress-induced myocardial ischemia after myocardial infarction suggests potential interactions among psychosocial stress, microvascular dysfunction, hormonal status and immune activation, but adequately powered mechanistic studies are still lacking (Rooks et al., 2016; Vaccarino et al., 2018). In addition, brain-heart communication is recursive rather than linear: depression may increase CHD risk, while myocardial infarction may precipitate depression; inflammation may promote plaque progression, while plaque rupture may amplify systemic inflammation. Finally, clinical translation remains uncertain (Matter et al., 2024; Panoutsopoulou et al., 2026). It is unrealistic to measure autonomic function, inflammatory biomarkers, mental stress ischemia, cerebral perfusion, brain inflammation and extracellular vesicles in every patient. The key challenge is to identify clinically actionable phenotypes and match them to targeted interventions.

Future studies should move beyond descriptive association toward causal network testing. Longitudinal multimodal cohorts are needed to follow patients with stable CHD or recent myocardial infarction using coronary imaging, autonomic markers, inflammatory profiling, mental health assessment, sleep and circadian measures, cognitive testing and brain imaging (Hammadah et al., 2017b; Toval et al., 2024). Such designs would help define temporal sequences and feedback loops in brain-heart communication. Mental stress testing should also be integrated with mechanistic biomarkers, including coronary flow assessment, endothelial function, catecholamines, inflammatory mediators and brain imaging, to identify distinct stress-response phenotypes. Human neuroimmune cardiovascular interfaces require deeper investigation, particularly plaque innervation, adventitial immune structures and artery-brain signaling pathways. Post-myocardial infarction neuroinflammation should be examined clinically using PET and MRI to determine whether brain inflammatory signals predict depression, cognitive decline, autonomic dysfunction or recurrent cardiovascular events. Extracellular vesicle research should prioritize reproducibility, tissue specificity and cargo characterization, especially distinguishing cardiac-derived vesicles from platelet-, endothelial- and immune-derived vesicles. Finally, intervention trials should apply brain–heart axis phenotyping. Rather than testing stress reduction, anti-inflammatory therapy or neuromodulation in unselected CHD populations, future trials should enrich for patients with measurable target engagement, such as mental stress-induced myocardial ischemia, low vagal tone, high inflammatory burden or post-infarction neuroinflammatory activation.

Coronary heart disease is not merely a disease of coronary narrowing; it is embedded in a bidirectional brain-heart communication system involving central autonomic networks, stress hormones, immune cells, vascular signaling, metabolism, sleep-circadian regulation and extracellular vesicles. The brain may influence coronary inflammation, vasomotion, microvascular function and ischemic susceptibility, while the diseased heart and coronary vasculature may signal back to the brain, contributing to neuroinflammation, altered interoception, depression, anxiety and cognitive vulnerability. This reciprocal framework may help explain why patients with similar coronary anatomy differ substantially in symptoms, stress responses, recovery trajectories and outcomes. Importantly, a brain-heart axis perspective does not replace established CHD therapies such as lipid lowering, antithrombotic treatment, revascularization or blood pressure control. Instead, it adds a systems-level layer to prevention and treatment. The next step is to define actionable phenotypes of maladaptive brain–heart communication and test targeted interventions. For selected patients, future CHD care may require not only stabilizing plaques and improving myocardial perfusion, but also restoring healthier communication between coronary tissue and the brain.

## Supplementary material

Supplementary Figure 1 Flow diagram of literature identification and selection. Records were identified through PubMed/MEDLINE, Embase, Web of Science, and reference-list screening. After duplicate removal, titles and abstracts were screened, followed by full-text eligibility assessment. A total of 93 studies were included in the final narrative synthesis.
